# Novel Roles for Notch3 and Notch4 Receptors in Gene Expression and Susceptibility to Ozone-Induced Lung Inflammation in Mice

**DOI:** 10.1289/ehp.1408852

**Published:** 2015-02-06

**Authors:** Kirsten C. Verhein, Zachary McCaw, Wesley Gladwell, Shweta Trivedi, Pierre R. Bushel, Steven R. Kleeberger

**Affiliations:** 1 Laboratory of Respiratory Biology, National Institute of Environmental Health Sciences (NIEHS), National Institutes of Health (NIH), Department of Health and Human Resources (DHHS), Research Triangle Park, North Carolina, USA; 2 Department of Animal Science, North Carolina State University, Raleigh, North Carolina, USA; 3 Biostatistics Branch, NIEHS, NIH, DHHS, Research Triangle Park, North Carolina, USA

## Abstract

**Background:**

Ozone is a highly toxic air pollutant and global health concern. Mechanisms of genetic susceptibility to ozone-induced lung inflammation are not completely understood. We hypothesized that *Notch3* and *Notch4* are important determinants of susceptibility to ozone-induced lung inflammation.

**Methods:**

Wild-type (WT), *Notch3* (*Notch3^–/–^*), and *Notch4* (*Notch4^–/–^*) knockout mice were exposed to ozone (0.3 ppm) or filtered air for 6–72 hr.

**Results:**

Relative to air-exposed controls, ozone increased bronchoalveolar lavage fluid (BALF) protein, a marker of lung permeability, in all genotypes, but significantly greater concentrations were found in *Notch4^–/–^* compared with WT and *Notch3^–/–^* mice. Significantly greater mean numbers of BALF neutrophils were found in *Notch3^–/–^* and *Notch4^–/–^* mice compared with WT mice after ozone exposure. Expression of whole lung *Tnf* was significantly increased after ozone in *Notch3^–/–^* and *Notch4^–/–^* mice, and was significantly greater in *Notch3^–/–^* compared with WT mice. Statistical analyses of the transcriptome identified differentially expressed gene networks between WT and knockout mice basally and after ozone, and included *Trim30*, a member of the inflammasome pathway, and *Traf6*, an inflammatory signaling member.

**Conclusions:**

These novel findings are consistent with *Notch3* and *Notch4* as susceptibility genes for ozone-induced lung injury, and suggest that Notch receptors protect against innate immune inflammation.

**Citation:**

Verhein KC, McCaw Z, Gladwell W, Trivedi S, Bushel PR, Kleeberger SR. 2015. Novel roles for Notch3 and Notch4 receptors in gene expression and susceptibility to ozone-induced lung inflammation in mice. Environ Health Perspect 123:799–805; http://dx.doi.org/10.1289/ehp.1408852

## Introduction

Ozone is a global, highly toxic air pollutant and principal component of smog. Elevated ambient ozone is associated with increased hospitalizations and exacerbation of respiratory conditions (Bell et al. 2004; Tatum and Shapiro 2005). Numerous components of the innate immune response are altered as a result of ozone toxicity in human and rodent airways, including predominant neutrophilic inflammation and airway hyperresponsiveness, chemokine and cytokine production, damage to airway epithelium, and increased mucus production and secretion (Al-Hegelan et al. 2011; Hollingsworth et al. 2007; Oakes et al. 2013). Ozone interacts initially with airway epithelial cells, alveolar macrophages, and epithelial lining fluid to initiate induction of cytokines and chemokines [including tumor necrosis factor (TNF-α), interleukin (IL)-6, IL-8, IL-1β, prostaglandins, and granulocyte-macrophage colony-stimulating factor (GM-CSF)] that recruit innate immune cells such as neutrophils. Interindividual (e.g., Alexeeff et al. 2008; Chen et al. 2007; McDonnell 1991; Yang et al. 2008) and interstrain (e.g., Bauer et al. 2010; Hamade et al. 2010; Kleeberger et al. 1997; Prows et al. 1997; Vancza et al. 2009) variation in lung responses to ozone have been reported, but mechanisms of susceptibility are not completely understood.

We used positional cloning to identify candidate ozone-susceptibility genes in a significant quantitative trait locus (QTL) on mouse chromosome 7 (*Inf2*) (Bauer et al. 2010; Kleeberger et al. 1997), including histocompatibility genes and *Tnf,* which are involved in innate immune function. TNF-α is a key inflammatory cytokine that contributes to ozone-induced pulmonary inflammation (Cho et al. 2007). In humans, an activating polymorphism in *Tnf* enhances susceptibility to ozone and asthma (Lee et al. 2009). Another candidate susceptibility gene in *Inf2* is *Notch4*, and just proximal to *Inf2* is *Notch3*. Notch receptors are evolutionarily conserved cell surface receptors important for cell fate decisions and embryonic development (Osborne and Minter 2007). In the lung, Notch4 is primarily expressed by endothelial cells, and Notch3 is expressed throughout (Post et al. 2000). Recent evidence suggests that Notch signaling is important in a mouse model of allergic asthma. Expression of Notch ligand Jagged1 on dendritic cells is induced by lipopolysaccharide (LPS) and is important for development of allergen-induced airway inflammation (Okamoto et al. 2012). Kang et al. (2009) inhibited Notch signaling with a γ-secretase inhibitor, which prevented antigen-induced airway inflammation in mice. In the present investigation, we hypothesized that Notch3 and Notch 4 receptors are critical to lung innate immune inflammatory response to ozone, and in particular the neutrophilic influx into the lung.

## Materials and Methods

*Animals and inhalation exposure*. Male *Notch3*^–/–^ (B6;129S1-*Notch3^tm1Grid^*/J), *Notch4*^–/–^ (B6;129S1-*Notch4^tm1Grid^*/J), and wild-type (WT) mice (B6129SF1/J; 7–13 weeks of age; average body weight, 22.6 ± 0.3 g) were purchased from Jackson Laboratories. Males were chosen for this study because we used males in our previous study to identify the *Inf2* QTL on chromosome 17 and because we wished to avoid potential confounders such as sex in the present study (Bauer et al. 2010; Kleeberger et al. 1997). Experimental groups had between 3 and 10 mice per group (details for individual experiments are included in the figure legends). Mice were at the exposure facility for approximately 1 week before they were acclimated for 2–4 days in individual stainless-steel wire cages in a Hazelton 1000 exposure chamber (Lab Products) containing a charcoal and HEPA-filtered air supply. Mice had access to food (NIH-07 chow; Zeigler Brothers) and water *ad libitum.* Mice were exposed continuously to 0.3 ppm ozone for 6, 24, 48, or 72 hr as previously described (Cho et al. 2007), and parallel exposures to filtered air were performed in a separate chamber for the same duration. Ozone was generated with a silent arc discharge ozone generator (model L-11; Pacific Ozone Technology) using ultra-high-purity air (National Welders Inc.). Chamber air temperature (72 ± 3°F) and humidity (50 ± 15%) were held constant. The ozone concentration was monitored and varied < 1% over the duration of exposure (Dasibi model 1008-PC; Dasibi Environmental Corp.). Animals were treated humanely and with regard to alleviation of suffering. All animal use was approved by the National Institute of Environmental Health Sciences Animal Care and Use Committee.

*Etanercept treatment*. Mice were treated with etanercept [Enbrel, 10 mg/kg, intraperitoneal (i.p.) injection; Immunex] or vehicle (human IgG, 10 mg/kg; Abcam) 18 hr before exposure to 0.3 ppm ozone for 24 hr (Budinger et al. 2011). The dose of etanercept was chosen to ensure inhibition of TNF-α signaling in the mice for the duration of the ozone exposure (Budinger et al. 2011). Drugs were diluted in sterile phosphate-buffered saline (PBS).

*Phenotype analysis*. Immediately after exposures, animals were killed with sodium pentobarbital (104 mg/kg, i.p.). The right lungs only were lavaged four times with Hanks’ balanced salt solution (17.5 mL/kg, pH 7.2–7.4). Lavage fluid was centrifuged, and supernatant from the first lavage was assayed for total protein concentration (a marker of lung permeability) using the Bradford assay (Thermo Scientific). Cell pellets were pooled, resuspended in Hanks’ balanced salt solution, and counted with a hemocytometer. Cells were spun onto glass slides and stained with Wright-Giesma stain (Diff-Quik; Baxter Scientific Products) for differential cell count analysis.

*Nuclear protein extraction and nuclear factor-kappaB (NF-*κ*B) activation*. Nuclear protein was isolated from frozen left-lung homogenates using a Nuclear Extract Kit (Active Motif) according to manufacturers instructions. Protein concentration was measured using the *DC* protein assay (Bio-Rad Laboratories), and nuclear protein from three animals per genotype/exposure group (air, 24-, and 48-hr ozone exposure) was equally pooled to achieve 10 μg of nuclear protein per NF-κB assay well. NF-κB activation was measured using a p65 transcription factor assay kit (TransAM p65; Active Motif). Samples were measured in duplicate, and the assay was repeated three times.

*Total lung RNA isolation and quantitative reverse-transcriptase polymerase chain reaction (qRT-PCR)*. Total RNA was isolated from frozen left-lung homogenates using the RNeasy Mini Plus Kit (QIAGEN) following the manufacturer’s instructions. One microgram of RNA was reverse transcribed into cDNA using MuLV reverse transcriptase (Life Technologies) at 42°C for 15 min and 95°C for 5 min with a Gene Amp PCR system 9700 (Applied Biosystems). Real-time qPCR was performed on a 1-μL sample of cDNA with either TaqMan probes or SybrGreen (both from Life Technologies) using a StepOnePlus Real-Time PCR System (Applied Biosystems, Life Technologies) and the following Taqman Probes: β*-Actin*, Mm00607939_s1; *Tnf*, Mm00443260_g1; *Notch1*, Mm00435249_m1; *Notch2*, Mm00803077_m1; *Notch3*, Mm00435270_m1; *Jag1*, Mm00496902_m1; *Jag2*, Mm01325629_m1; *Dll1*, Mm01279269_m1; *Hes1*, Mm01342805_m1; *Cxcl2*, Mm00436450_m1. Primers for *Notch4* were as follows: forward, GGA GAC TGC AGA CCA GAA GG; reverse, GAC CCT CAG AGT CAG GGA CA. Quantification of amplified products was performed using the ΔΔCt method with β-actin as the internal control.

*Microarray analysis of lung transcriptome*. Total whole lung RNA was used after passing quality control using an Agilent Bioanalyzer 2100 (Agilent Technologies Inc.). GeneChip analysis (Affymetrix) on the Affymetrix Mouse Genome 430 2.0 array containing > 39,000 transcripts was performed on each treatment group in triplicate. Expression intensity values were normalized with the robust multiarray average (RMA) method using GeneSpring GX 11.0 Expression Analysis Software (Agilent Technologies). Transcripts were further analyzed by two-way analysis of variance (ANOVA) with Tukey’s honest significant difference (HSD) post hoc tests comparing genotype (WT, *Notch3^–/–^, Notch4^–/–^*) and exposure (air, 6 hr ozone, 24 hr ozone, 48 hr ozone). Genes were determined to be statistically differentially expressed at the Benjamini-Hochberg multiple testing correction adjusted *p*-value < 0.05. Gene lists were analyzed using Ingenuity Pathway Analysis (QIAGEN). Microarray data were submitted to CEBS (Chemical Effects in Biological Systems; 005-00003-0112-000-3) and GEO (Gene Expression Omnibus; GSE58244).

*EPIG (extracting microarray gene expression patterns and identifying coexpressed genes) pattern analysis*. We used EPIG to characterize gene expression patterns (Chou et al. 2007). The approach utilizes the underlying structure of gene expression data to extract patterns and identify coexpressed genes that are responsive to experimental conditions. For each genotype group, the RMA-normalized data for each probe set was converted to a ratio by dividing the probe set pixel intensity by the average of its respective time-matched air control samples from that group and then log base 2 transforming the ratio. EPIG uses Pearson correlation (*r*) across all the nine groups of samples, signal-to-noise (*s*/*n*) ratio within groups of samples, and magnitude of fold change (FC) for a probe set within a group to first detect all potential patterns in the data and coexpressed gene probe sets detected in terms of statistically significant (*p*-value < 10^–4^) correlation between the probe set profiles and the pattern. The parameter settings for the EPIG analysis were the defaults: *r* = 0.8, *s*/*n* = 2.5, and FC = 0.5. We used a minimum pattern cluster size of six for finding all potential patterns.

*Statistics*. All data are expressed as group mean ± SE. Two-way ANOVA was used to evaluate effects of ozone on pulmonary injury in WT and knockout mice. Factors in this analysis were exposure (air or ozone) and genotype (WT, *Notch3^–/–^*, or *Notch4^–/–^*). Data from air-exposed mice were pooled because there was no postexposure time effect on bronchoalveolar lavage fluid (BALF) protein concentration or cells. All statistical analyses were performed using GraphPad Prism, version 6.0 (GraphPad Software).

## Results and Discussion

*Targeted deletion of* Notch3 *and* Notch4 *enhances susceptibility to ozone-induced lung inflammation*. To assess the role of *Notch3* and *Notch4* in ozone-induced lung inflammation, we continuously exposed WT mice and mice with targeted deletion of *Notch3* (*Notch3^–/–^*) or *Notch4* (*Notch4^–/–^*) to ozone and measured markers of lung inflammation and injury. In mice, sub-acute exposure to 0.3 ppm ozone represents an environmentally relevant dosing protocol that elicits airway inflammation that has been reported previously (e.g., Backus et al. 2010; Cho et al. 2007; Johansson et al. 2010; Kasahara et al. 2012). This dosing protocol was chosen for the present study because it led to the initial discovery of the *Inf2* QTL containing ozone susceptibility genes in mice (Bauer and Kleeberger 2010; Kleeberger et al. 1997).

**Figure 1:**
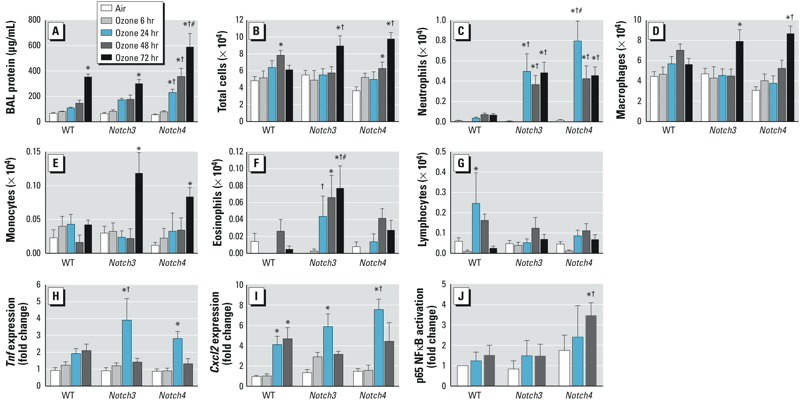
*Notch3^–/–^* and *Notch4^–/–^* mice are more susceptible to ozone-induced lung inflammation compared with WT mice. (*A*) Bronchoalveolar lavage fluid (BALF) protein concentration increased significantly after 72 hr ozone in all mice; protein concentration increased earliest (24 hr) and most significantly in *Notch4^–/–^* mice. (*B*) BALF total cells increased in WT mice after 48 hr and in *Notch3^–/–^* and *Notch4^–/–^* mice after 72 hr. (*C*) Neutrophils significantly increased in *Notch3^–/–^* and *Notch4^–/–^* mice after 24–72 hr compared with air-exposed and WT mice. BAL macrophages (*D*) and monocytes (*E*) significantly increased in* Notch3^–/–^* and *Notch4^–/–^* mice after 72 hr. (*F*) BAL eosinophils significantly increased only in *Notch3^–/–^* mice. (*G*) BAL lymphocytes significantly increased only in WT mice after 24 hr. (*H*) Expression of *Tnf* in whole-lung homogenates significantly increased in *Notch3^–/–^* and *Notch4^–/–^* mice after 24 hr. (*I*) Expression of *Cxcl2* in whole-lung homogenates increased in all groups after 24 hr; *n* = 4–10 mice per group (exposed in 2–3 groups). In *A–I*, *p < 0.05 compared with respective air controls, ^†^*p* < 0.05 compared with WT at same time point, and ^#^*p* < 0.05 between *Notch3^–/–^* and *Notch4^–/–^* mice at the same time point, by 2-way ANOVA with Bonferroni post hoc tests. (*J*) p65 NF-κB activation was significantly increased after 48 hr ozone in *Notch4^–/–^* mice and when compared with WT and *Notch3^–/–^* mice. *p < 0.05 compared with respective air control, and ^†^*p* < 0.05 compared with WT and *Notch3^–/–^* ozone, by 2-way ANOVA with Holm-Sidak pairwise comparisons.

**Figure 2:**
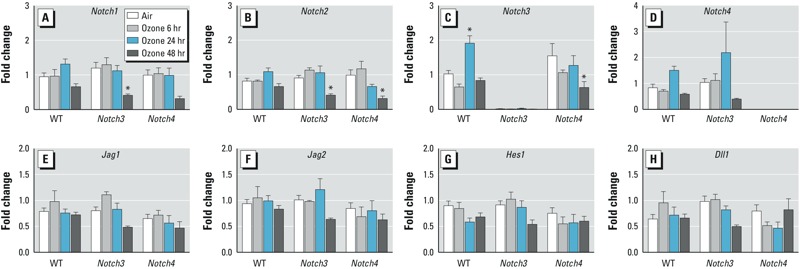
Real-time PCR of whole-lung homogenates for Notch receptors, ligands, and target genes. (*A*) *Notch1*. (*B*) *Notch2*. (*C*) *Notch3*. (*D*) *Notch4*. (*E*) *Jag1*. (*F*) *Jag2*. (*G*) *Hes1*. (*H*) *Dll1*. Expression of *Notch1* and *Notch2* decreased in *Notch3^–/–^* and *Notch4^–/–^* mice after 48 hr exposure to ozone. *n* = 3–10 mice per group (exposed in 2–3 groups). *p < 0.05 compared with respective air controls, by 2-way ANOVA with Bonferroni post hoc tests.

Although significantly increased compared with air controls, inflammation parameters (BALF protein and total cells) in WT mice were minimal after ozone exposure (Figure 1A,B) compared with more susceptible strains (Cho et al. 2001). Relative to air-exposed controls, deletion of either *Notch3* or *Notch4* markedly enhanced ozone-induced increases in the numbers of macrophages and monocytes in BALF (Figure 1D,E; see also Supplemental Material, Table S1) and whole-lung expression of proinflammatory mediators *Tnf* and *Cxcl2* (macrophage inflammatory protein 2-alpha; *Mip2*) (Figure 1H,I). Lymphocytes in BALF were not consistently changed by ozone exposure; a significant increase was found only after 24-hr ozone exposure in WT mice (Figure 1G). We also measured an increase in NF-κB activation after 48-hr ozone in *Notch4^–/–^* mice (Figure 1J). Neither *Notch3* nor *Notch4* were expressed in animals with their respective deletion (Figure 2C,D), and we found no ozone or genotype effect on known Notch ligands (*Jagged1*, *Jagged2*, and *Dll1*) or the effector molecule *Hes1* (Figure 2E–H).

The most notable change in BALF inflammatory markers was found for neutrophils. Highly significant increases were found in *Notch3^–/–^* and *Notch4^–/–^* mice compared with their respective air controls and WT mice (Figure 1C). Moreover, ozone-induced changes in BALF protein, a marker for lung injury, were significantly increased in *Notch4^–/–^* mice relative to the other genotypes (Figure 1A). These results suggest a protective role for *Notch* in ozone-induced lung injury and inflammation.

**Figure 3:**
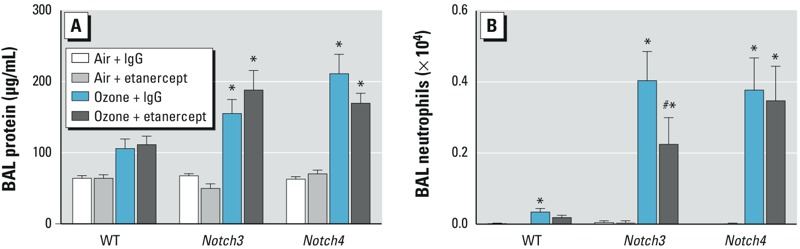
Blocking TNF signaling with etanercept reduced airway neutrophilia in *Notch3* knockout mice. Prior to 24‑hr ozone exposure, mice were treated with a TNF inhibitor (etanercept, 10 mg/kg i.p.) or vehicle (human IgG, 10 mg/kg i.p.). (*A*) Etanercept pretreatment did not affect BALF protein concentration. (*B*) Etanercept reduced the ozone-induced influx of neutrophils in *Notch3^–/–^* mice. *n* = 5–10 mice per group. *p < 0.05 compared with respective air controls, and ^#^*p* < 0.05 between etanercept and vehicle after ozone exposure, by 2-way ANOVA with Tukey’s post hoc tests.

A potential mechanism through which Notch3 and Notch4 protect against ozone-induced inflammation is by modulation of *Tnf* expression. TNF-α is increased during ozone-induced airway inflammation, and TNF inhibition reduces neutrophilia (Cho et al. 2001; Shore et al. 2001). To determine whether TNF-α mediates exacerbated inflammation in *Notch*-deficient mice, we treated WT, *Notch3^–/–^*, and *Notch4^–/–^* mice with the TNF-α inhibitor etanercept before ozone exposure. Etanercept had no effect on BALF protein concentration (Figure 3A), as predicted because antibodies to TNF, as well as targeted deletion of TNF receptors and *Tnf*, also had no effect on lung permeability (Cho et al. 2001). In WT mice, neutrophils were significantly increased in BALF after ozone exposure with IgG/vehicle treatment and were not significantly increased after etanercept treatment (Figure 3B). However, in *Notch3^–/–^* mice, etanercept significantly reduced neutrophils compared with IgG controls (*p* = 0.042) but had no effect on neutrophil influx in *Notch4^–/–^*, even though *Tnf* gene expression was increased in these mice (Figure 3B). Our results therefore support a TNF-α–mediated role for Notch3 but not Notch4 in protection against ozone-induced inflammation, and suggest that the mechanism behind increased susceptibility is different for mice with targeted deletion of *Notch3* and *Notch4*. The link between TNF-α and Notch signaling likely occurs through NF-κB in a cell type– and Notch receptor– specific manner. Positive and negative regulation of NF-κB by Notch receptors has been demonstrated previously. For example, Notch1 and Notch2 negatively regulate Toll-like receptor (TLR) signaling in macrophages by inhibiting NF-κB transcription activity (Zhang et al. 2012). In contrast, Notch1 signaling activates NF-κB by suppressing an inhibitor of IκB kinase in T-cell acute lymphoblastic leukemia cells (Espinosa et al. 2010). TNF-α also regulates Notch signaling. In rheumatoid synovial fibroblasts, TNF-α stimulation induced expression of *Notch1* and *Notch4* and nuclear translocation of the intracellular domain (Ando et al. 2003). Additional investigations are necessary to understand the specific mechanistic interaction of TNF-α and Notch3 signaling in response to ozone.

Eosinophils were also significantly increased after ozone exposure, but only in *Notch3^–/–^* mice (Figure 1F). Currently there is no evidence that eosinophils express Notch3 receptors, but both human and mouse eosinophils have been shown to express Notch1 and Notch2 receptors that control eosinophil differentiation (Kang et al. 2007; Radke et al. 2009). In the present study the numbers of eosinophils counted after ozone exposure was very small, and although significant, it is unclear whether the increase is biologically relevant. Eosinophils are known to cluster around airway nerves following ozone exposure, so it is possible that eosinophils are recruited to the lung by ozone but the majority remain in the tissue and do not migrate to the alveoli (Yost et al. 2005). Further investigation will be required to test this hypothesis.

**Figure 4:**
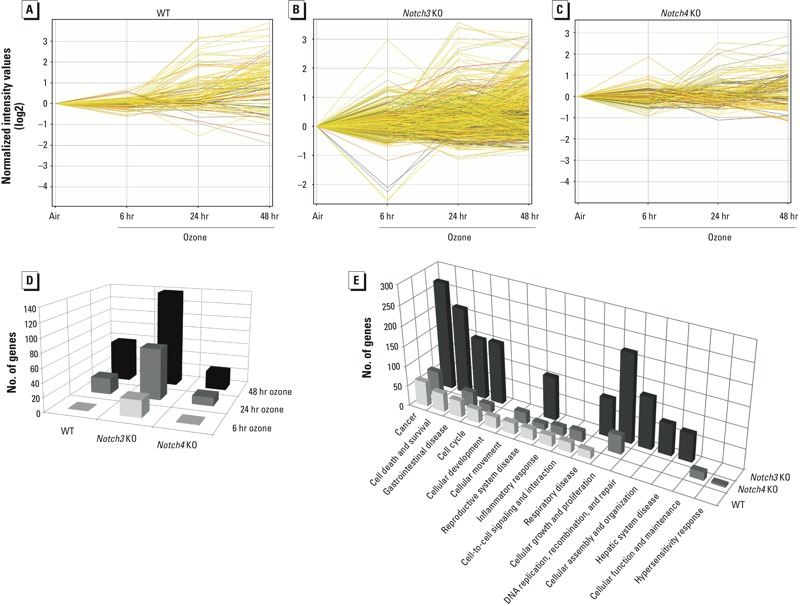
Gene expression microarray from whole-lung homogenates identified differentially expressed genes after exposure to ozone. KO, knockout. (*A*) In WT mice, 116 transcripts were differentially expressed at at least one time point after exposure to ozone compared with air. (*B*) In *Notch3^–/–^* mice, 739 transcripts were differentially expressed at at least one time point after ozone compared with air. (*C*) In *Notch4^–/–^* mice, 155 transcripts were differentially expressed at at least one time point after ozone compared with air. *p* < 0.05, ANOVA with Tukey’s HSD post hoc test and Benjamini-Hochberg multiple testing correction. (*D*) Number of significantly differentially expressed genes after ozone compared with air. (*E*) Top 10 significant biological functions from Ingenuity Pathway Analysis and numbers of differentially expressed genes in each category.

*Transcriptomic analysis identified candidate gene networks for Notch-mediated lung inflammation*. Although TNF-α may influence a portion of Notch3-mediated protection against ozone-induced inflammation, additional mechanisms must contribute to the effect in mice with deletion of *Notch3* and *Notch4*. To further understand the downstream pathways through which *Notch3* and *Notch4* mediate ozone-induced lung inflammation, we used genome-wide transcriptome analysis to identify candidate gene transcripts and interactomes. ANOVA modeling of the data identified few differentially expressed genes between WT and knockout mice after air exposure (see Supplemental Material, Table S2). However, ANOVA and pattern recognition EPIG (Chou et al. 2007) analyses identified ozone-induced changes in many gene transcripts in all genotypes (Figure 4A–C; see also Supplemental Material, Tables S2–S4).

To identify differential gene expression after ozone, we normalized transcripts to air exposure within each genotype. In WT and *Notch4^–/–^* mice, ANOVA modeling found the majority of differentially expressed gene transcripts after 24–48 hr ozone (Figure 4D). Across all time points, the greatest number of ozone-responsive transcripts was found in *Notch3^–/–^* mice (Figure 4D). Relative to WT, seven biological function categories identified by Ingenuity Pathway Analysis were similar in *Notch4^–/–^* mice and three (cellular growth and proliferation, cellular function and maintenance, and hypersensitivity response) were unique to *Notch4^–/–^* (Figure 4E; see also Supplemental Material, Table S3). The biological function categories in *Notch3^–/–^* mice diverged from WT and *Notch4^–/–^*. Although six categories were in common with WT and *Notch3^–/–^*, three categories (DNA replication, recombination, and repair; cellular assembly and organization; and hepatic system disease) were unique to *Notch3^–/–^* mice (Figure 4E; see also Supplemental Material, Table S3). Although the inflammatory cell phenotypes of *Notch3^–/–^* and *Notch4^–/–^* mice were nearly identical, the greater number of differentially expressed transcripts in each biological function, and the greater diversity of transcripts in *Notch3^–/–^* mice are consistent with the hypothesis that mechanisms for protective effects of *Notch3* against ozone-induced inflammation differ from those of *Notch4*.

**Figure 5:**
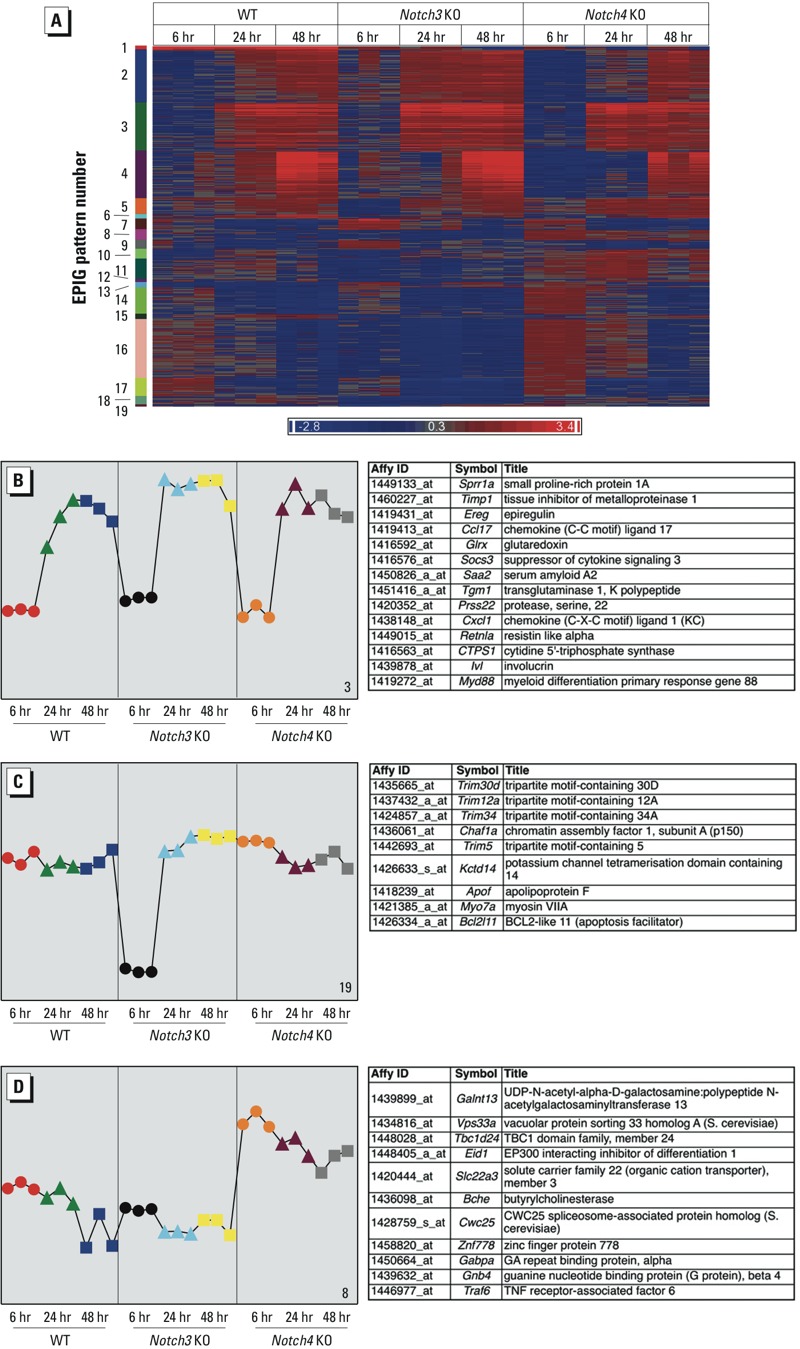
Pattern analysis of transcript expression using EPIG (extracting microarray gene expression patterns and identifying coexpressed genes)–identified candidate genes. KO, knockout. (*A*) Heat map of 1,723 transcripts that fit in 19 patterns (labeled numerically) identified by EPIG (see Supplemental Material, Table S4, for full list of genes in all patterns). Red and blue colors correspond to up‑ and down-regulated genes, respectively. Transcripts are colored by fold change relative to respective air controls. (*B*) Pattern 3 represents transcripts that increased after 24 (triangles) or 48 (squares) hr ozone exposure in all genotypes (232 transcripts; top 14 most significant are shown on right). (*C*) Pattern 19 represents transcripts that decreased after 6 hr (circles) ozone only in *Notch3^–/–^* mice (12 transcripts, top 9 shown on right). (*D*) Pattern 8 represents transcripts that change only in *Notch4^–/–^* mice. These transcripts increased after ozone at all time points (50 transcripts, top 11 shown on right). In (*B–D*), the colors of symbols******represent the nine groups of samples, and profiles represent the average of the top 6 gene probe set profiles within the respective pattern. The *y*‑axes indicate the changes in gene expression (log2 intensity) relative to air-exposed controls.

EPIG was used to further understand the expression patterns that underlie the mechanistically different protective effects of *Notch3* and *Notch4*. After normalization to respective air-exposed controls, EPIG identified in WT, *Notch3^–/–^*, and *Notch4^–/–^* mice 19 distinct patterns of gene expression with 1,723 coexpressed transcripts (Figure 5A; see also Supplemental Material, Figure S1). The number of transcripts in each pattern ranged from 12 (pattern 19) to 281 (pattern 16) (see Supplemental Material, Table S4).

We prioritized further analysis of three transcript patterns to profile ozone-responsive transcripts common to all three genotypes (pattern 3), or unique to *Notch3* and *Notch4* (patterns 19 and 8) (Figure 5B–5D). Pattern 3 consisted of transcripts that increased after 24 and 48 hr ozone in all mice (Figure 5B; see also Supplemental Material, Table S4). The top significant gene ontology (GO) enrichment categories of these ozone-responsive genes included G protein-coupled receptor binding, chemokine and cytokine activity, and chemokine receptor binding (see Supplemental Material, Table S4). Included in pattern 3 are genes known to be associated with ozone-induced inflammation, such as *Socs3* (Backus et al. 2010), *Myd88* (Bauer et al. 2011), and *Cxcl1* (Lazaar et al. 2011).

Pattern 19 profiles transcripts that decreased after 6 hr ozone only in *Notch3^–/–^* mice (Figure 5C). These *Notch3^–/–^*–specific transcripts included tripartite motif containing genes (Trim) *Trim30*, *Trim12a*, *Trim5*, and *Trim6-Trim34*. Trim30 was recently described as a negative regulator of the inflammasome (Hu et al. 2010), a protein complex important for release of mature inflammatory cytokines IL-1β and IL-18 that is activated by a number of stimuli, including oxidant stress. Ozone causes oxidative stress, and Feng et al. (2012) showed that targeted deletion of various inflammasome components protects against ozone-induced lung inflammation. Thus, decreased expression of *Trim30* specifically in *Notch3^–/–^* mice may lead to increased inflammasome activation and enhanced ozone-induced lung inflammation.

Pattern 8 consisted of transcripts that increased after ozone exposure at all time points only in *Notch4^–/–^* mice (Figure 5D). These transcripts are significantly enriched for GO category positive regulation of immune system processes (see Supplemental Material, Table S4); half of the identified genes in pattern 8 are expressed by at least one type of inflammatory cell and nine genes are expressed by stem cells (BioGPS database; http://www.biogps.org). Transcripts included TNF receptor-associated factor 6 (*Traf6*), which is a unique member of the TNF receptor-associated factor family that signals through the IL-1 receptor and TLR superfamily and is not activated by the TNF receptor. Activation of TRAF6 induces NF-κB through IκB kinase (Deng et al. 2000). Increased expression of *Traf6* in *Notch4^–/–^* mice may explain the increase in NF-κB activation seen in these mice 48 hr after ozone exposure (Figure 1J). The role of TRAF6 in ozone-induced lung inflammation has not been identified; however, IL-1 and TLR receptors are known to be important. Therefore, *Notch4* deficiency may enhance ozone-induced inflammation by increasing *Traf6* expression, thereby increasing activation of NF-κB and the inflammatory sequelae.

Notch receptors and their ligands are known to induce differentiation of T-cell subsets (Amsen et al. 2004). Our gene expression analyses indicate that the absence of *Notch3* or *Notch4* caused differentially expressed genes involved in T-cell signaling. In *Notch4^–/–^* mice after ozone exposure, *Zap70* was up-regulated (see Supplemental Material, Table S4). Zap70 is part of the T-cell receptor and is crucial for TCR signaling and development of CD8^+^ cells (Schim van der Loeff et al. 2014). In *Notch3^–/–^* mice, the ANOVA analysis indicated that *Il33* was up-regulated after 24 hr exposure to ozone, an inducer of Th2 cytokines (see Supplemental Material, Table S2). Therefore, it is possible that the absence of Notch3 or Notch4 signaling affects T-cell differentiation or development. Future studies will be necessary to characterize specific T-cell populations present before and after ozone exposure in mice with *Notch3* and *Notch4* deletion.

## Conclusion

We tested the hypothesis that *Inf2* cluster genes *Notch3* and *Notch4* contribute to ozone-induced lung inflammation. Enhanced inflammation in *Notch3^–/–^* and *Notch4^–/–^* mice compared with WT mice supports a novel role for Notch 3 and 4 receptors in modulating innate immune lung inflammatory responses to ozone. Moreover, genome-wide transcriptomic analyses suggest that these receptors protect against inflammation through different mechanisms. Future investigations should provide greater insight to the role of innate immunity in differential susceptibility to oxidant injury.

## Supplemental Material

(645 KB) PDF
