# Supplementary material for: Novel Roles for Notch3 and Notch4 Receptors in Gene Expression and Susceptibility to Ozone-Induced Lung Inflammation in Mice
Source: Environ Health Perspect. 2015 Feb 6;123(8):799–805. doi: 10.1289/ehp.1408852 (PMC4529014; doi:10.1289/ehp.1408852)
Supplement: (645 KB) PDF [file ehp.1408852.s001.508.pdf]

Supplemental Material

Novel Roles for Notch3 and Notch4 Receptors in Gene Expression and Susceptibility to Ozone-Induced Lung Inflammation in Mice

Kirsten C. Verhein, Zachary McCaw, Wesley Gladwell, Shweta Trivedi, Pierre R. Bushel, and Steven R. Kleeberger

Table of Contents

Figure S1. EPIG analysis thumbnail images Page 3

Table S1. Bronchoalveolar lavage phenotype data Page 4

Additional Excel Files

Supplemental Material, Table S2. ANOVA analysis results

This Excel file includes 4 separate tables in individual worksheets:

- Supplemental Table S2, WT.N3.N4 Air. Genes identified by ANOVA that are significantly differentially expressed in air-exposed mice of all genotypes.
- Supplemental Table S2, WT Air vs Ozone. Genes identified by ANOVA that are significantly differentially expressed in wild type mice after ozone.
- Supplemental Table S2, Notch3 KO Air vs Ozone. Genes identified by ANOVA that are significantly differentially expressed in *Notch3*$^{-/-}$ mice after ozone.
- Supplemental Table S2, Notch4 KO Air vs Ozone. Genes identified by ANOVA that are significantly differentially expressed in *Notch4*$^{-/-}$ mice after ozone.

Supplemental Material, Table S3. Ingenuity Pathway Analysis gene list functions.

This Excel file includes 4 separate tables in individual worksheets:

- Supplemental Material, Table S3, WT. IPA functions in wild-type mice exposed to ozone from genes significant via ANOVA analysis.
- Supplemental Material, Table S3, Notch 3. IPA functions in *Notch3*$^{-/-}$ mice exposed to ozone from genes significant via ANOVA analysis.
- Supplemental Material, Table S3, Notch 4. IPA functions in *Notch4*$^{-/-}$ mice exposed to ozone.
- Supplemental Material, Table S3, Intersections. IPA functions and genes in common between WT, *Notch3*$^{-/-}$, and *Notch4*$^{-/-}$.

Supplemental Material, Table S4. EPIG expression pattern results.

EPIG analyses for wild type, *Notch3*$^{-/-}$, and *Notch4*$^{-/-}$ mice exposed to ozone.

This Excel file includes 21 separate tables in individual worksheets:

- Supplemental Table S4, Total EPIG Output. Genes and statistics for all identified patterns.
- Supplemental Table S4, GO Enrichment for EPIG patterns.
- Supplemental Table S4, Pattern 1–Pattern 19: Gene lists for each of the 19 patterns identified by the EPIG analyses.

Supplemental Material, Figure S1

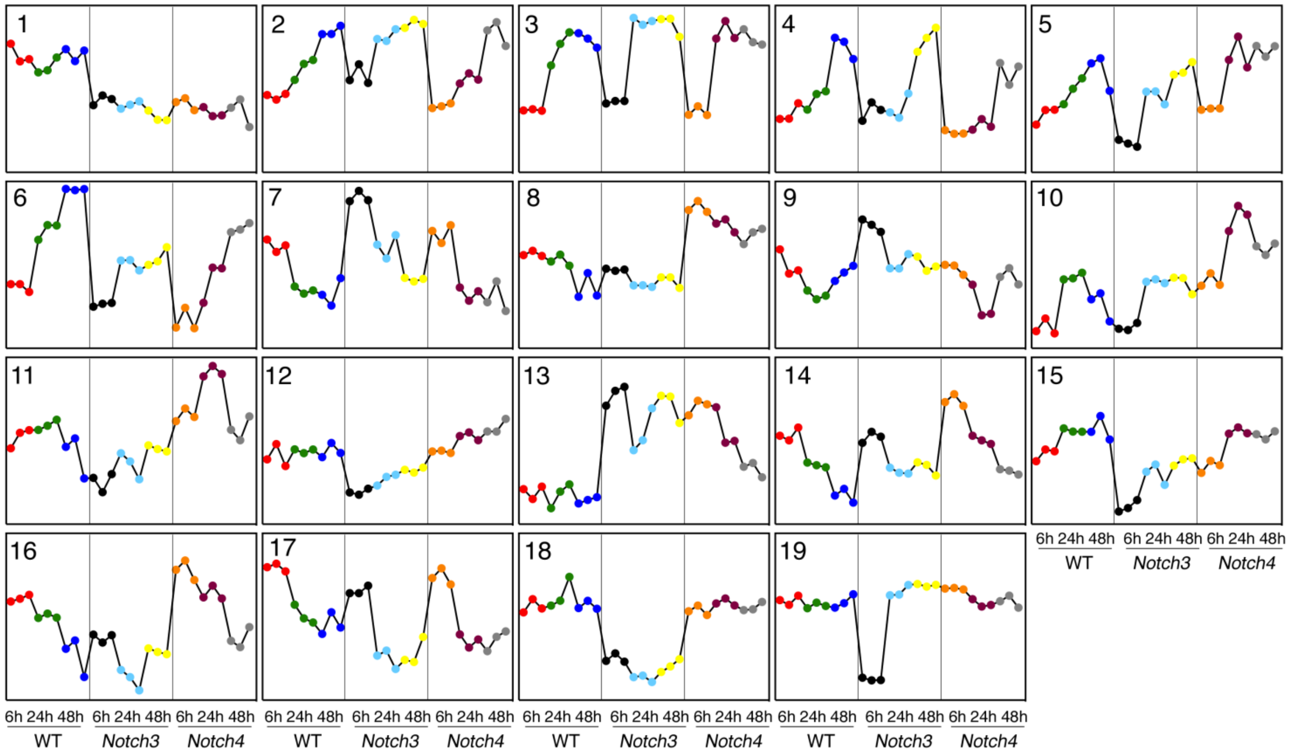

Supplemental Material, Figure S1. Patterns extracted by EPIG from transcripts after ozone exposure in wild type and knockout mice. 19 patterns were generated from 1723 differentially expressed transcripts. The vertical axes are the changes in gene expression (log2 intensity) relative to air-exposed controls. Colors are used to help distinguish data for each exposure time. Exposure times are also indicated on the x-axis for each panel.

Supplemental Material, Table S1. Bronchoalveolar lavage phenotype data.

Sample	Air	6h Ozone	24h Ozone	48h Ozone	72h Ozone
**Protein**					
WT	65.8 ± 6.6 (10)	80.7 ± 3.3 (4)	109.0 ± 6.1 (10)	145.6 ± 24.5 (7)	354.3 ± 22.3* (8)
Notch3 KO	64.4 ± 9.6 (10)	82.6 ±11.4 (4)	172.6 ± 12.2 (8)	176.9 ± 33.4 (6)	301.5 ± 30.7* (7)
Notch4 KO	55.8 ± 5.1 (10)	79.0 ±7.8 (4)	230.4 ± 25.7*† (8)	356.1 ± 64.3*† (7)	589.7 ± 104.6*† (8)
**Total Cells**					
WT	4.8 ± 0.5 (10)	5.2 ± 0.8 (4)	6.4 ± 0.8 (10)	7.8 ± 0.6* (8)	6.1 ± 0.5 (8)
Notch3 KO	5.5 ± 0.5 (9)	4.9 ± 1.1 (4)	5.5 ± 0.7 (8)	5.8 ± 0.7 (6)	9.0 ± 1.2*† (7)
Notch4 KO	3.6 ± 0.5 (9)	5.2 ± 0.5 (4)	5.0 ± 0.9 (8)	6.3 ± 0.8* (7)	9.8 ± 0.7*† (8)
**Macrophages**					
WT	4.4 ± 0.5 (10)	4.7 ± 0.7 (4)	5.7 ± 0.7 (10)	7.0 ± 0.6 (8)	5.6 ± 0.6 (8)
Notch3 KO	4.7 ± 0.5 (10)	4.3 ± 1.1 (4)	4.5 ± 0.7 (8)	4.5 ± 0.7 (5)	7.9 ± 1.1* (7)
Notch4 KO	3.1 ± 0.4 (10)	4.0 ± 0.7 (4)	3.8 ± 0.7 (8)	5.2 ± 0.8 (7)	8.7 ± 0.7*† (8)
**Neutrophils**					
WT	0.01 ± 0.0 (13)	0.0 ± 0.0 (4)	0.04 ± 0.01 (10)	0.07 ± 0.01 (14)	0.07 ± 0.01 (8)
Notch3 KO	0.01 ± 0.0 (10)	0.0 ± 0.0 (4)	0.50 ± 0.17*†, (8)	0.37 ± 0.09*† (5)	0.48 ± 0.10*† (7)
Notch4 KO	0.01 ± 0.01 (11)	0.0 ± 0.0 (4)	0.80 ± 0.20*† (8)	0.42 ± 0.13*† (7)	0.46 ± 0.08*†# (8)
**Epithelial Cells**					
WT	0.32 ± 0.08 (10)	0.45 ± 0.10 (4)	0.38 ± 0.06 (10)	0.59 ± 0.20 (8)	0.36 ± 0.11 (8)
Notch3 KO	0.47 ± 0.09 (10)	0.54 ± 0.12 (4)	0.36 ± 0.05 (8)	0.46 ± 0.09 (5)	0.30 ± 0.03 (7)
Notch4 KO	0.46 ± 0.07 (10)	0.49 ± 0.08 (4)	0.29 ± 0.02 (8)	0.42 ± 0.06 (7)	0.47 ± 0.09 (8)
**Eosinophils**					
WT	0.01 ± 0.01 (10)	0.0 ± 0.0 (4)	0.0 ± 0.0 (10)	0.03 ± 0.01 (8)	0.01 ± 0.00 (8)
Notch3 KO	0.0 ± 0.0 (10)	0.0 ± 0.0 (4)	0.04 ± 0.02† (8)	0.07 ± 0.03* (5)	0.08 ± 0.03*†# (7)
Notch4 KO	0.01 ± 0.01 (10)	0.0 ± 0.0 (4)	0.01 ± 0.01 (8)	0.04 ± 0.01 (7)	0.03 ± 0.01 (8)
**Lymphocytes**					
WT	0.06 ± 0.02 (10)	0.01 ± 0.01 (4)	0.25 ± 0.15* (10)	0.16 ± 0.03 (8)	0.03 ± 0.01 (8)
Notch3 KO	0.05 ± 0.02 (10)	0.04 ± 0.01 (4)	0.05 ± 0.02 (8)	0.12 ± 0.05 (5)	0.07 ± 0.02 (7)
Notch4 KO	0.05 ± 0.01 (10)	0.01 ± 0.00 (4)	0.09 ± 0.03 (8)	0.11 ± 0.03 (7)	0.07 ± 0.02 (8)
**Monocytes**					
WT	0.02 ± 0.01 (10)	0.04 ± 0.01 (4)	0.04 ± 0.01 (10)	0.02 ± 0.01 (8)	0.04 ± 0.01 (8)
Notch3 KO	0.03 ± 0.01 (10)	0.03 ± 0.01 (4)	0.02 ± 0.01 (8)	0.02 ± 0.01 (5)	0.12 ± 0.03* (7)
Notch4 KO	0.01 ± 0.00 (10)	0.02 ± 0.01 (4)	0.03 ± 0.03 (8)	0.03 ± 0.02 (7)	0.08 ± 0.01* (8)

Data are presented as mean ± SEM. Sample sizes are in parentheses. Protein is µg/ml, and cell data are x10^4/ml. Data were analyzed with 2-way ANOVA and Bonferroni post-hoc tests. *$P < 0.05$ compared to respective air controls; †$P < 0.05$ compared to WT at same time point; #$P < 0.05$ between *Notch3*$^{-/-}$ and *Notch4*$^{-/-}$ mice at the same time point.
